# Synthesis and Characterization of PEDOT:P(SS-*co*-VTMS) with Hydrophobic Properties and Excellent Thermal Stability

**DOI:** 10.3390/polym8050189

**Published:** 2016-05-12

**Authors:** Wonseok Cho, Soeun Im, Seyul Kim, Soyeon Kim, Jung Hyun Kim

**Affiliations:** Department of Chemical and Biomolecular Engineering, Yonsei University, 134 Shinchon-Dong, Seodaemoon-Gu, Seoul 120-749, Korea; intersv@yonsei.ac.kr (W.C.); imse218@yonsei.ac.kr (S.I.); kimseyul84@yonsei.ac.kr (Se.K.); kimso88@yonsei.ac.kr (So.K.)

**Keywords:** PEDOT:PSS, conducting polymer, PSS copolymer

## Abstract

Hydrophobic and comparatively thermally-stable poly(3,4-ethylenedioxythiophene), *i.e.*, poly(styrene sulfonate-*co*-vinyltrimethoxysilane) (PEDOT:P(SS-*co*-VTMS)) copolymer was successfully synthesized via the introduction of silane coupling agent into the PSS main chain to form P(SS-*co*-VTMS) copolymers. PSS and P(SS-*co*-VMTS) copolymers were successfully synthesized via radical solution polymerization, and PEDOT:P(SS-*co*-VTMS) was synthesized via Fe^+^-catalyzed oxidative polymerization. The characterization of PEDOT:P(SS-*co*-VTMS) was performed through an analysis of Fourier transform infrared spectroscopy (FTIR) results, water contact angle and optical images. The electrical properties of conductive PEDOT:P(SS-*co*-VTMS) thin films were evaluated by studying the influence of the VTMS content on the electrical and physical properties. The conductivity of PEDOT:P(SS-*co*-VTMS) decreased with an increase in the VTMS content, but was close to that of the PEDOT:PSS, 235.9 S·cm^−1^. The introduction of VTMS into the PSS copolymer improved the mechanical properties and thermal stability and increased the hydrophobicity. The thermal stability test at a temperature over 240 °C indicated that the sheet resistance of PEDOT:PSS increased by 3,012%. The sheet resistance of PEDOT:P(SS-*co*-VTMS), on the other hand, only increased by 480%. The stability of PEDOT:P(SS-*co*-VTMS) was six-times higher than that of the reference PEDOT:PSS.

## 1. Introduction

Conductive polymers (CPs) have attracted considerable attention for use in various potential applications, owing to their excellent properties, such as high conductivity, optical and electrical properties and processability. The unique properties of the CPs have provided the materials with an almost unlimited range of applicability [1,2,3,4,5,6,7,8,9]. The CPs showed excellent physical and electrical properties, which can easily be manipulated as in many previous studies. 3,4-ethylenedioxy thiophene (PEDOT) is one of the CPs with such excellent properties. However, the introduction of polystyrene sulfonic acid (PSS) to solve the main problem of PEDOT, which is its insolubility in water and solvents, limits the applicability, owing to the intrinsic disadvantages of the PSS polymers, such as their low stability in water, chemicals and certain weather conditions. Because of the stability problem, the conductivity of PEDOT:PSS films decreases rapidly at high temperatures and/or high humidity [10,11,12]. To improve the water and weather stabilities of PEDOT:PSS, some research groups proposed the introduction of agents, such as silane, organic monomer and polymers. However, the introduction of another material, especially an insulating one, into PEDOT:PSS can negatively influence the electrical and physical properties of conducting films and limit and the number of potential applications.

To overcome the problems related to PSS, a few materials have been introduced into the PEDOT:PSS, thereby improving the electrical and physical properties and the stability of the materials. In previous studies, two possible methods were attempted. One was to replace the PSS with a stabilizer, and the other was to introduce functional groups into the PSS main chains. Replacing PSS can dramatically increase the stability of the PEDOT materials; however, the electrical properties deteriorate. For example, sulfonated poly(imide) used as a template for PEDOT to increase the thermal stability decreased the conductivity below 10 S·cm^−1^ [13,14,15]. Poly(styrene)-poly(butyl acrylate) silica was also used as a template for PEDOT, but the product did not have stability, and silica materials were needed as a coupling agent [16,17]. Introducing some functional groups, self-crosslink polymers in particular, to simplify the process, into the PSS chains can be easily controlled via radical polymerization with materials possessing the vinyl or acryl groups. The modified PSS copolymers with high stability in water have already been used in fuel cells. The imide bond of *N*-methyl acrylamide (NMA) was also introduced into the PSS main chain. The product had a higher stability than the pristine PEDOT:PSS; however, the conductivity of films decreased by more than 50% [10].

In this paper, silane-coupling crosslinkable groups were incorporated into 4-styrene sulfonic acid, sodium salt (NaSS), to synthesize the random copolymer, P(SS-*co*-vinyltrimethoxysilane (VTMS)). VTMS is one of the silane coupling agents and can be polymerized with their vinyl functional groups being processed as the monomer with styrene via radical polymerization. In spite of the high water solubility of NaSS, the copolymer of P(SS-*co*-VTMS) showed relatively low solubility because of the hydrophobic groups of VTMS. However, the copolymer had higher stability in water and, thus, proved to be a possible water-soluble template for PEDOT synthesis. Without any crosslinking agent, the two functional groups in PSS and VTMS could react with each other with drying at high temperatures. Two possible crosslinking mechanisms were proposed: one is the silane coupling mechanism and the other is the reaction of the –SO_3_^−^ terminal of PSS and the –OH terminal of VTMS with the release of H_2_O molecules with thermal curing [18]. The random copolymer P(SS-*co*-VTMS) could readily be synthesized via aqueous solution polymerization. Then, the obtained P(SS-*co*-VTMS) was used as a polymeric template during oxidative polymerization of 3,4-ethylenedioxythiophene (EDOT), and the product, namely, PEDOT:P(SS-*co*-VTMS), in conductive dispersion showed colloidal stability. The thermally-curable PEDOT:P(SS-*co*-VTMS) conductive films not only had good conductivity and transparency, but also showed some excellent performances, such as higher hydrophobicity and thermal stability.

## 2. Materials and Methods

### 2.1. Materials

For synthesis of conductive PEDOT:P(SS-*co*-VTMS), the monomers of P(SS-*co*-VTMS) copolymers, *i.e.*, styrene sulfonic acid, sodium salt hydrate (NaSS, 328,596, >4.2% of water) and vinyltrimethoxysilane (VTMS, 235,768, >98%), the PEDOT monomer 3,4-ethylenedioxythiophene (EDOT, 483,028, 97%) and the initiators, *i.e.*, sodium persulfate (NaPS) and iron(III) sulfate pentahydrate (Fe_2_(SO_4_)_3_·5H_2_O), were purchased from Sigma Aldrich Co., Yongin-si, Gyeonggi-do, Korea. For the fabrication of conductive polymer films, PEDOT:P(SS-*co*-VTMS) was used as synthesized. Dimethyl sulfoxide (DMSO) was purchased from Sigma Aldrich Co., Yongin-Si, Gyeonggi-do, Korea. Positive ion exchanger (TRILITE SAR) and base-ion exchanger (TRILITE UPW) were purchased from Samyang Co., Seongnam-si, Gyeonggi-do, Korea. All materials were used as received without any further purification. Double-distilled and deionized (DDI) water was used throughout the work.

### 2.2. Preparation of P(SS-co-VTMS) Copolymer and PEDOT:P(SS-co-VTMS)

A series of PSS polymers and P(SS-*co*-VTMS) copolymers was prepared via solution radical polymerization. The molar ratio of VTMS in P(SS-*co*-VTMS) copolymer was varied in the range from 0–15 mol% (VTMS 0 mol %, 5 mol %, 10 mol %, 15 mol %). The molar ratio of VTMS was calculated by the following equation.

(1) $$\text{Molar content of VTMS in copolymer}\;\left(\text{mol}\%\right)=\;\frac{\frac{\text{Weight of VTMS}}{M_{w}\;\text{of VTMS}}}{\frac{\text{Weight of VTMS}}{M_{w}\;\text{of VTMS}}+\frac{\text{Weight of NaSS}}{M_{w}\;\text{of NaSS}}}\times 100$$

The procedure of polymerization is as follows. First, NaSS and VTMS were stabilized in 100 mL of DDI water at 80 °C for 10 min. Then, 0.5 g of NaPS in a solution state was added as the initiator, and then, the polymerization was performed at 80 °C for 12 h. After the completion of the polymerization, products were ion-exchanged to remove any residue and by-products of initiators, by using 26 mg of positive ion exchangers for 2 h at room temperature.

A series of PEDOT:P(SS-*co*-VTMS) were polymerized via oxidative polymerization, using the series of P(SS-*co*-VTMS) described as above (VTMS 0 mol %, 5 mol %, 10 mol %, 15 mol%). The emulsion was prepared in a 100-mL four-necked double-jacketed glass reactor equipped with a mechanical stirrer. First, 0.84 g of P(SS-*co*-VTMS) was stabilized in 80 g of DDI water under argon purge with stirring at 300 rpm. The amount of P(SS-*co*-VTMS) copolymer was adjusted to match with 2.5 mass equivalents of EDOT monomer. Then, 0.772 g of NaSS and 0.0053 g of Fe_2_(SO_4_)_3_ were added to the reactor in series, and then, the EDOT monomer was added for initiation. The mixture was maintained for 23 h under full argon purge conditions. After the polymerization completed, the products were ion-exchanged to remove the residue and by-products of initiators, by using 31 mg of positive ion exchangers and 25 mg of negative ion exchangers for 2 h at room temperature.

### 2.3. Characterization of P(SS-co-VTMS) and PEDOT:P(SS-co-VTMS)

The structural characterization of P(SS-*co*-VTMS) copolymers was done by FTIR (Bruker, Vortex 70, Seongnam-si, Korea) in the range 2,000–400 cm^−1^ with a resolution of 2 cm and nuclear magnetic resonance (NMR) (Bruker Biospin, Avance II, Seongnam-si, Korea) with a 400-MHz frequency. The samples for FTIR and NMR were dried in a vacuum oven at 150 °C with thermal curing. The FTIR analysis was performed on the powder state of P(SS-*co*-VTMS), mixed with KBr powder and palletized. A transmittance mode was used to obtain the FTIR absorption spectrum. The NMR analysis failed due to the insolubility of PSS copolymers. The thermal properties of solid-state copolymers were studied via a thermal gravimetric analyzer (Perkin Elmer, STA800, Seoul, Korea) at temperatures ranging from room temperature to 800 °C. To analyze the water contact angle, the PSS and P(SS-*co*-VTMS) films were fabricated via spin-coating on pre-treated glass substrates, which was followed by drying in the oven at 150 °C for 5 min.

For the preparation of the conductive films, PEDOT:PSS and PEDOT:P(SS-*co*-VTMS) were spin coated on the pre-treated glass substrates at 500 rpm for 40 s and were dried in the oven at 150 °C for 5 min. Before the formation of the film, the DMSO was mixed into the solution and was stabilized at least for 1 h. The sheet resistances were measured by the four-point probe method (Napson, RT-70V/RG-5, Seongnam-si, Gyeonggi-do, Korea), and the film thicknesses were measured with the surface profiler (KLA-Tencor Co., Alpha step 500 Surface Profiler (AS500), Milpitas, CA, United States); the average of values corresponding to least 10 points for each sample was calculated. The transmittance values at 550 nm and absorption values between 350 and 2000 nm were obtained by a UV–VIS–NIR spectrophotometer (Agilent, Cary5000, Santa Clara, CA, United States). The references of all transmittance values were bare glass. The elemental compositions of the PEDOT:PSS and PEDOT:P(SS-*co*-VTMS) film surface were analyzed by X-ray photoelectron spectroscopy (XPS, Thermo Fisher Scientific Co., K-Alpha, Seoul, Korea) using monochromated Al Kα X-ray radiation. The phase image of films was analyzed with atomic force microscope (AFM, Park Systems Co., XE-100, Suwon-si, Gyeonggio-do, Korea).

## 3. Results and Discussions

### 3.1. The Synthesis and Characterization of P(SS-co-VTMS) Copolymers

**Figure 1:**
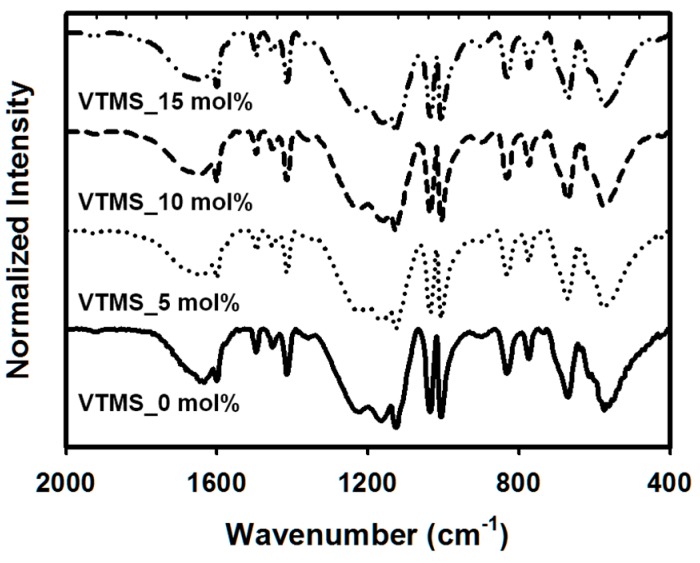
FTIR spectra of PSS and P(SS-*co*-vinyltrimethoxysilane (VTMS)) copolymers.

In this study, the silane coupling agent was introduced into the PSS copolymer chains to improve the thermal stability and hydrophobicity of the films. To control the molar ratio of the introduced silane groups, the FTIR spectra of P(SS-*co*-VTMS) copolymers in the solid state and subjected to thermal annealing were studied. As shown in Figure 1, all of the spectra of PSS and P(SS-*co*-VTMS) showed the vibration bands at 1,640 cm^−1^ and between 1,495 and 1,412 cm^−1^, relative to the skeletal vibration of C=C in the aromatic ring. The vibration bands between 1,250 and 1,140 cm^−1^ with relatively broad shoulder peaks were derived from SO_3_ asymmetric stretching vibration, and the bands at 1,040 and 1,010 cm^−1^ with relatively sharp peaks were derived from SO_3_ symmetric stretching vibration. These peaks mentioned above could be seen in the spectra of both PSS and P(SS-*co*-VTMS) copolymers, meaning that all of the copolymers had the –SO_3_ groups [10,19,20]. The VTMS-incorporated PSS showed a decrease in the peak intensity at 1100 cm^−1^, which is characteristic of the stretching vibration of Si–OH. As the VTMS molar ratio of the sample increased, a decrease in the peak intensity at 1080 cm^−1^, corresponding to the asymmetric stretching vibration of Si–O–Si, was observed [21,22]. Despite the almost complete overlap of peaks corresponding to PSS and PVTMS, the additional bands corresponding to VTMS can ensure copolymerization between NaSS and VTMS.

**Figure 2:**
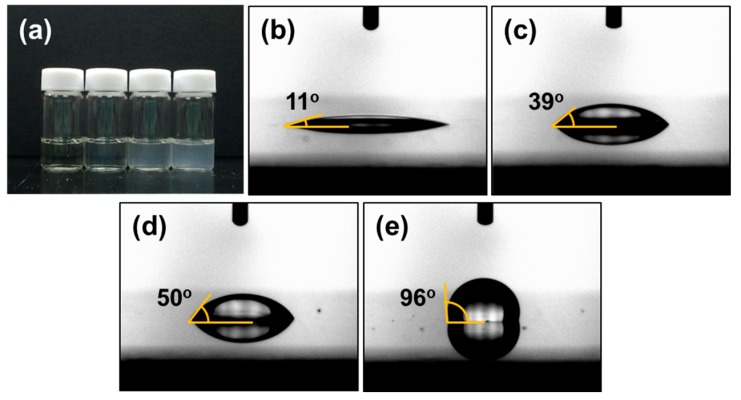
(**a**) Optical images of P(SS-*co*-VTMS) solutions and water contact angle images of PSS and P(SS-*co*-VTMS) films; (**b**) VTMS 0 mol%; (**c**) VTMS 5 mol%; (**d**) VTMS 10 mol%; (**e**) VTMS 15 mol%.

The hydrophobicity of VTMS can have an influence on both the aqueous solution and the hydrophobicity of dried films, as shown in Figure 2. As the VTMS molar ratio was increased, the synthesized polymer solution changed from transparent to translucent, as its properties changed from the solution phase to the emulsion phase. The PSS changed from hydrophilic to amphiphilic with the introduction of hydrophobic VTMS. Furthermore, the ^1^H– and ^13^C–NMR spectra were analyzed to comprehend the copolymerization mechanism. However, NMR analysis could not be performed because of the decrease in solubility following the copolymerization with VTMS. Similar to the symptoms of solution states, as the VTMS content was increased, the water contact angle on the copolymers dramatically increased from 11°–96°. After polymerization of P(SS-*co*-VTMS), the VTMS molecules were hydrolyzed to form active –OH groups under ion exchange treatment (IET) and could react with other VTMS active sites or –SO^3−^ of PSS to form interconnecting networks [18,22]. Therefore, based on these results, we could confirm that the VTMS content in the copolymers increased as the VTMS feed ratio was increased, and complete polymerization was achieved. Furthermore, in this study, the hydrophobicity of the PSS copolymer was considered to be the confirmation of copolymerization. The resultant outstanding changes in the hydrophobicity can lead to applicability in a variety of areas.

### 3.2. Effect of Introduction of VTMS into the PSS Main Chain on the Physical and Electrical Properties of PEDOT:P(SS-co-VTMS) Conductive Films

To explore the electrical and physical properties of the synthesized PEDOT:PSS and PEDOT:P(SS-*co*-VTMS) materials, conductive films were fabricated by spin coating with 5 wt % DMSO added to enhance the electrical properties. The mechanism of the conductivity enhancement at PEDOT:PSS, through treatments with solvents or materials, such as DMSO, has been extensively studied previously [1,3,4]. Here, it was found that the conductivity of films decreased as the VTMS content was increased. To explain this phenomenon, three analysis methods, widely used in conductive polymer film research, were used.

**Figure 3:**
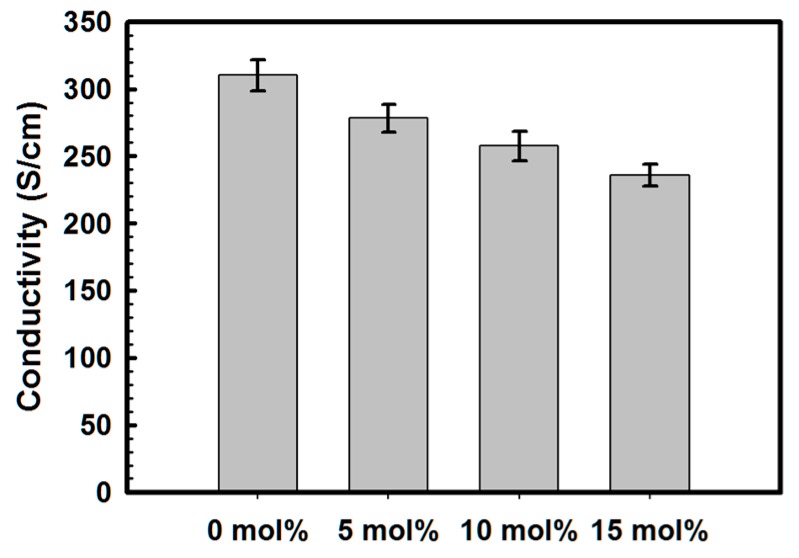
Conductivity of PEDOT:P(SS-*co*-VTMS) with DMSO solution treatment.

The conductivities of the synthesized polymers were calculated using the sheet resistance and thickness of the films. As shown in Figure 3, the conductivity of the films decreases linearly from 310.1 S/cm at PEDOT:PSS to 235.9 S/cm at PEDOT:P(SS-*co*-VTMS) with 15 mol % VTMS. Although the conductivity decrease was not high, it was observed that the introduction of VTMS into the PSS main chain affected the conductivity and other electrical properties, either through changes in its polymerization status or due to the influence on film formations. For increasing the conductivity of PEDOT:PSS, there are few things that should be discussed. The conductivity of PEDOT:PSS could be controlled not only by the intrinsic property of the polymer itself under controlling growth conditions, but also the post-treatment of PEDOT:PSS [1,23,24]. As a result, the electrical properties should be analyzed with both points mentioned above.

As a result, to explain the conductivity decrease, the PEDOT:PSS should be analyzed with three possible mechanisms that could affect the above two conditions: retardation of the PEDOT growth during polymerization, doping level change because of the reducing contents of PSS and the deterioration of the DMSO treatment effect by the introduction of VTMS.

**Figure 4:**
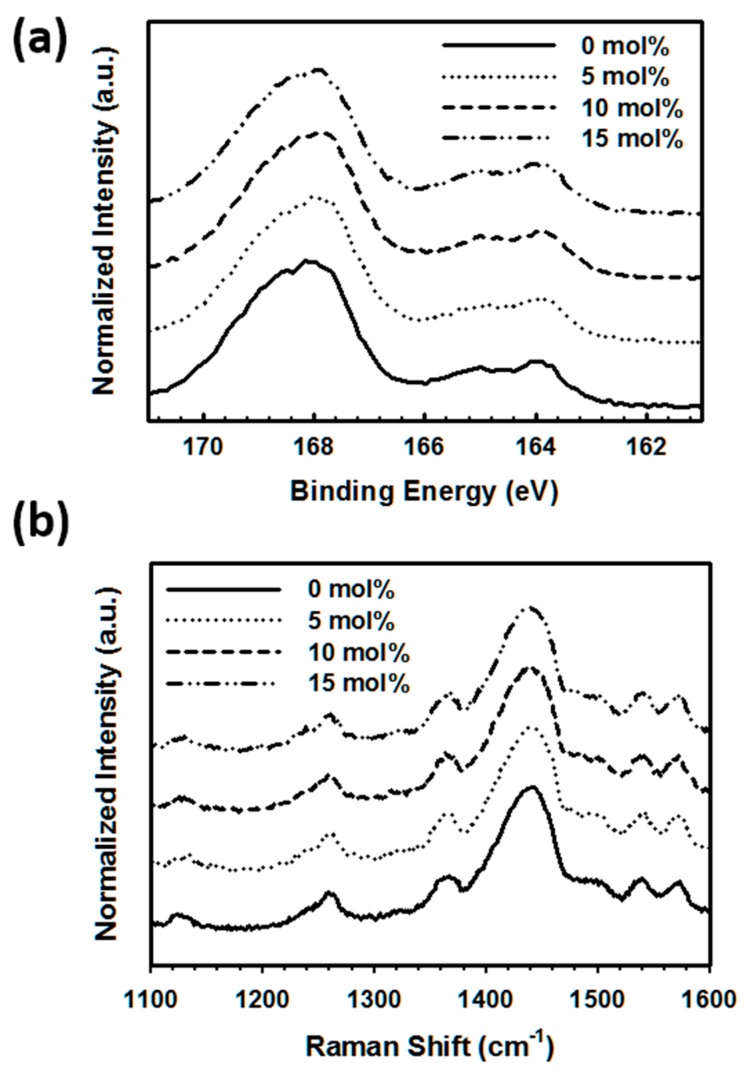
XPS spectra (**a**) and Raman shift spectra (**b**) of PEDOT:P(SS-*co*-VTMS).

First, the growth of the PEDOT polymerization was studied via XPS using the spin-coated PEDOT:P(SS-*co*-VTMS) films treated with DMSO. As shown in Figure 4a, the XPS S 2p spectra were almost the same at all PEDOT conditions. The S 2p peaks for sulfur atoms were obtained at 167.6 and 163.9 eV with PSS and PEDOT, respectively [5,25]. The residue and by-products, including any unreacted EDOT monomers, were already removed by IET purification following the PEDOT polymerization. As a result, if the PEDOT growth increased, the intensity of the peak at 163.9 eV would increase; however, the intensity of the peak at 163.9 eV remained constant for all of the samples. In detail, the peak intensity at 163.9 eV slightly increased as VTMS content was increased from 0–15 mol%, because as the ratio of PSS to PEDOT decreased following the increase in the VTMS content, the sulfur content in the copolymer also decreased. The calculated ratio almost correlated with the content ratios. As a result, it was concluded that the introduction of VTMS did not affect the polymerization of PEDOT.

To analyze the effects of changes in the doping level, the Raman spectra and UV–VIS near infrared (NIR) spectra were analyzed. As shown in Figure 4b, the Raman spectra were constant at all conditions. The Raman spectra shift to higher wavenumbers is generally induced by increased doping levels, as a consequence of the degree of backbone deformation during oxidation to polarons and bi-polarons and the associated transitions between benzoid- and quinoid-dominated forms. The strong band assigned to the symmetric *C*_α_=*C*_β_ stretching at 1,436 cm^−1^ indicates that the PEDOT had a quinoid structure, which did not change between the samples [5,20]. Similar to the Raman spectra, the UV–VIS–NIR spectra remained almost constant at all conditions (Figure S1). The findings are in agreement with the results of previous studies. The shape of the absorption spectrum of PEDOT:PSS was mostly dependent on the PEDOT doping level [6]. The primary doping level of PEDOT in each sample is independent of the introduction of VTMS into the copolymers. As a result, the primary doping level of PEDOT and PEDOT:P(SS-*co*-VTMS) did not change significantly.

**Figure 5:**
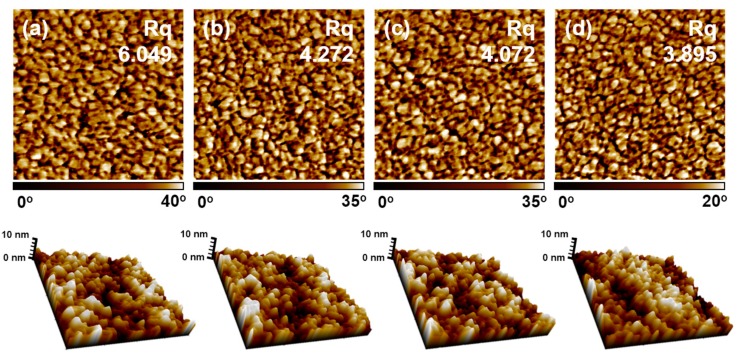
AFM phase images and topographic images of PEDOT:PSS and PEDOT:P(SS-*co*-VTMS) films with DMSO solution treatment. (**a**) PEDOT:P(SS-*co*-VTMS) 0 mol %; (**b**) PEDOT:P(SS-*co*-VTMS) 5 mol %; (**c**) PEDOT:P(SS-*co*-VTMS) 10 mol %; (**d**) PEDOT:P(SS-*co*-VTMS) 15 mol %.

For the confirmation of the last assumption, the AFM images were analyzed. Generally, the DMSO treatment increased the conductivity of PEDOT:PSS films through three possible mechanisms: phase separation between PEDOT and PSS, the swelling effect on the PEDOT domain and the reorientation of the PEDOT structure. For this study, all three mechanisms can be ignored because of the silane connection caused by VTMS. As shown in Figure 5, the phase image of PEDOT:P(SS-*co*-VTMS) can show the separation and structural arrangement between PEDOT and PSS: the bright (positive) and dark (negative) phase shifts correspond to PEDOT-rich grains and PSS-rich grains of PEDOT:PSS films [1]. We observed that the positive grains of the films were slightly decreased as the VTMS content was increased, as verified by the decreased Rq of the films, from 6.049 at PEDOT:PSS to 3.895 at PEDOT:P(SS-*co*-VTMS) at 15 mol %. In addition, the topographic images and roughness of the films did not show any differences, with similar good coating abilities. These findings were the same as previous research, showing the development of compact thin films structure, as well as PEDOT-rich granular networks, causing larger contact areas between oriented PEDOT grains. These results explained that the phase separation was highest with the pristine PSS and linearly decreased as VTMS content was increased, *i.e.*, smallest at VTMS 0 mol % and highest at VTMS 15 mol %, owing to the introduction of P(SS-co-VTMS).

We tried to validate the three assumptions that could correspond to the effects listed above: the first was that the conductivity change was derived from the growth retardation of PEDOT; the second was the decrease in the primary doping level; and the third was the decrease in the extent of the physical reorientation of PEDOT with DMSO post-treatment. Further studies can explain that the polymerization and level of doping were independent of the introduction of VTMS to the PSS, even at relatively high VTMS content. First of all, the doping levels were maintained at all conditions, studied by both the Raman shift and UV–VIS–NIR, without any changes. Secondly, no sort of retardation of PEDOT growth was shown in XPS studies. As a result, the conductivity decreased by the lowering of the enhancing effect on the PEDOT through crosslinking between the copolymers.

### 3.3. Practical Implications of the High Hydrophobicity and Thermal Stability of PEDOT:P(SS-co-VTMS)

**Figure 6:**
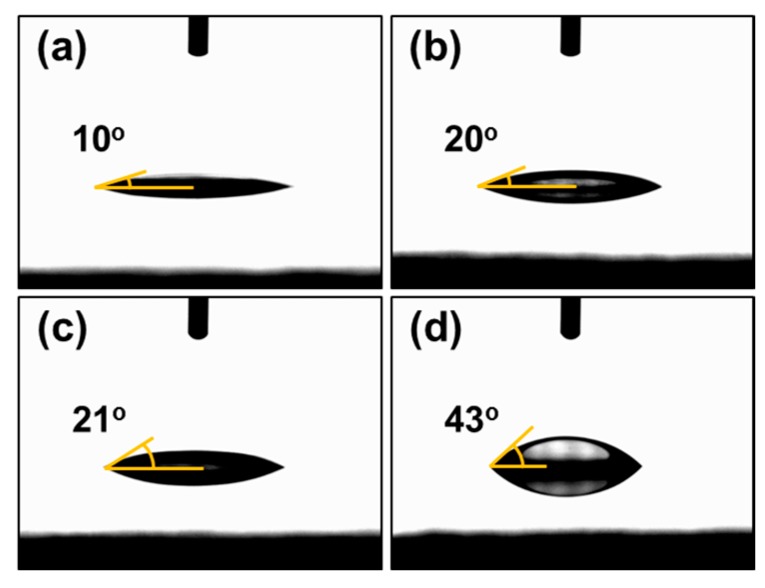
Water contact angle optical images of PEDOT:PSS and PEDOT:P(SS-*co*-VTMS) films with DMSO solution treatment. (**a**) PEDOT:P(SS-*co*-VTMS) 0 mol%; (**b**) PEDOT:P(SS-*co*-VTMS) 5 mol%; (**c**) PEDOT:P(SS-*co*-VTMS) 10 mol%; (**d**) PEDOT:P(SS-*co*-VTMS) 15 mol%.

The introduction of VTMS into the PSS main chain can improve the physical properties of the PEDOT:PSS films. The silane coupling agent can increase the hydrophobicity of the materials. As shown in Figure 6, not only the hydrophobicity of PSS copolymers, but also the hydrophobicity of PEDOT:PSS dramatically increased, from 11°–43°. The hydrophilicity of PSS causes it to be easily re-dispersed, lowering the product applicability. The increase in hydrophobicity without lowering the emulsion stability or affecting the electrical properties has not been reported previously. The hydrolyzed VTMS molecules affect the conductive films by reacting with other VTMS active sites or –SO^3−^ of PSS forming the interconnecting networks [18].

The introduction of silane coupling sites can increase both the hydrophobicity and the stability of materials. From the previous report, the symptom of thermal degradation, as a decrease of the conductivity and electrical properties, is derived from following mechanism [26]. The thermal degradation could be regarded as the corrosion of the PEDOT:PSS structure and conducting pathway, by reducing the size of the PEDOT grains and consequently increasing the distances between each PEDOT grains, equivalently increasing the potential barrier for polaron transport between PEDOT. The typical model of thermal effect on conductivity is the variable-range hopping (VRH) model.

(2) $$\sigma (T)=\sigma_{0}\text{exp}\left[-{\left(\frac{T_{0}}{T}\right)}^{\alpha }\right]$$

More specifically, in the VRH model of transport, the exponent α depends not only on the exponent of the density of states near the Fermi energy level, but also the number of hopping process dimensions D. The thermal degradation of PEDOT:PSS diminishes the PSS polymers, breaking the electrostatic bond between PSS and PEDOT and gradually leading to reeling off of the PSS chain. To simplify, the thermal degradation of PEDOT:PSS is derived from the degradation of PSS and the structure of PEDOT:PSS, simultaneously increasing the distances between each PEDOT grain. As a result, for increasing the thermal stability of PEDOT:PSS, the PSS structure should be maintained during thermal aging processes.

**Figure 7:**
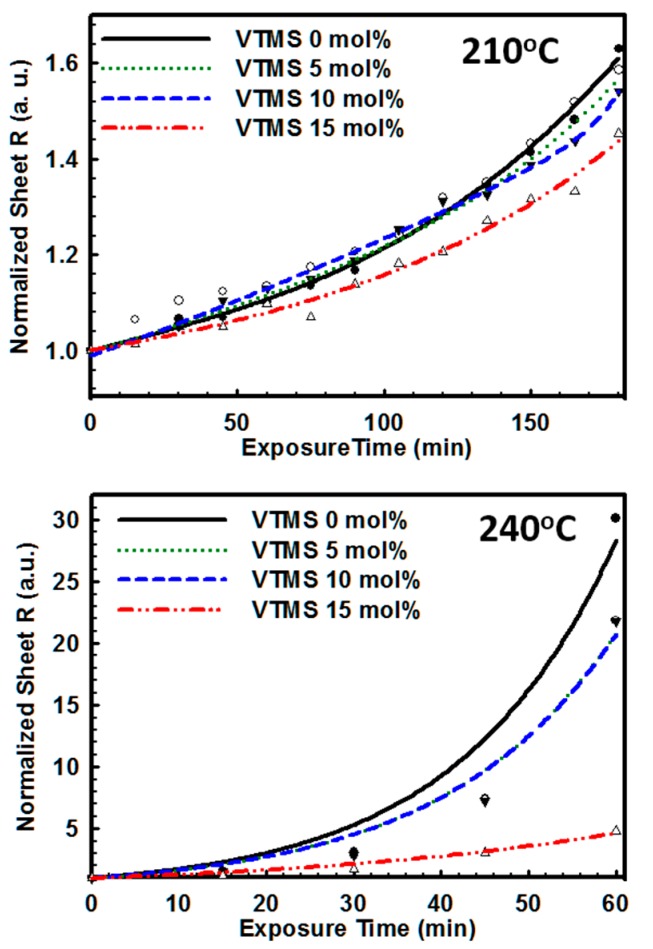
Thermal stability spectra of PEDOT:PSS and PEDOT:P(SS-*co*-VTMS) with the thermal exposure test.

To test the thermal stability of PEDOT films after the adoption of VTMS, the conductive films were exposed to high temperatures at 210 °C and 240 °C. For the aging test performed below 200 °C, it was observed that the films were stable without thermal damage, as indicated by a resistance increase. However, as shown in Figure 7, in the aging test performed above 200 °C, the sheet resistance of both the PEDOT:PSS and PEDOT:P(SS-*co*-VTMS) films significantly increased in only 2 h. For the thermal exposure test performed at 210 °C, the resistivity of films linearly increased by more than 50% in only 2 h. The increase in resistance was maximum for PEDOT:PSS and minimum for PEDOT:P(SS-*co*-VTMS) with 15 mol % VTMS. However, in the 210 °C test, a clearly distinguishable difference was not observed. To create more harsh conditions, the films were exposed to a thermal treatment at 240 °C. It was observed that the resistivity of the films exponentially increased. In this test, the differences between each sample could be measured, as changing from 480% at PEDOT:P(SS-*co*-VTMS) with 15 mol % VTMS to 3012% at PEDOT:PSS. The introduction of silane into the PSS chain resulted in a sharp increase in the thermal stability of PEDOT:PSS films. The thermal stability of the films was dramatically increased with the increase in the VTMS content, especially in harsh conditions, because the silane coupling agent increased the thermal stability of the PSS copolymer (Figure S2) [21]. Furthermore, after aging of the films, the ratio between PSS and PEDOT remained as the VTMS content increased in the P(SS-*co*-VTMS) conditions (Figure S3). As a result, the thermal stability increased with the introduction of VTMS, maintaining the structure of PSS relatively.

Although the thermal degradation of PEDOT:PSS could not be perfectly prevented by the introduction of VTMS, the possibility of a further stability increase exists.

## 4. Conclusions

We presented a method for the synthesis of conductive PEDOT:P(SS-*co*-VTMS) that exhibits hydrophobicity and thermal stability, using inorganic silane coupling P(SS-*co*-VTMS) copolymers. The FTIR and contact angle images of the PSS and P(SS-*co*-VTMS) confirmed the high conversion due to copolymerization. The introduction of VTMS into PSS influenced secondary doping through reorientation of PEDOT by DMSO treatment, which decreased the conductivity of PEDOT:PSS. Nevertheless, the introduction of VTMS to the PSS led to good stability and relatively high conductivity. Despite the decrease in conductivity at high molar contents of VTMS, PEDOT:P(SS-*co*-VTMS) showed improved hydrophobicity and high thermal stability. Furthermore, despite the changes in the properties of PEDOT:PSS due to the thermal effects that could not be perfectly controlled by using the VTMS copolymers, the improvement in thermal stability was clearly observed. As a result, we believe that the observed thermal stability and hydrophobicity of films in water will lead to the development of highly stable and conductive polymer films and will enhance their applicability at high temperatures in various fields.

## Supplementary Materials

Supplementary Materials can be found at www.mdpi.com/2073-4360/8/5/189/s1.
