# Supplementary material for: Synthesis and Characterization of PEDOT:P(SS-co-VTMS) with Hydrophobic Properties and Excellent Thermal Stability
Source: Polymers (Basel). 2016 May 12;8(5):189. doi: 10.3390/polym8050189 (PMC6431847; doi:10.3390/polym8050189)
Supplement: Supplementary file 1 [file polymers-08-00189-s001.pdf]

Supplementary Materials: Synthesis and Characterization of PEDOT:P(SS-*co*-VTMS) with Hydrophobic Properties and Excellent Thermal Stability

Wonseok Cho, Soeun Im, Seyul Kim, Soyeon Kim and Jung Hyun Kim

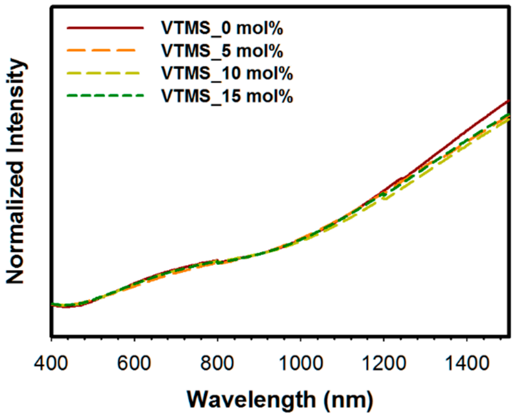

Figure S1. UV–VIS–NIR spectra of PEDOT:P(SS-*co*-VTMS) films with DMSO treatment.

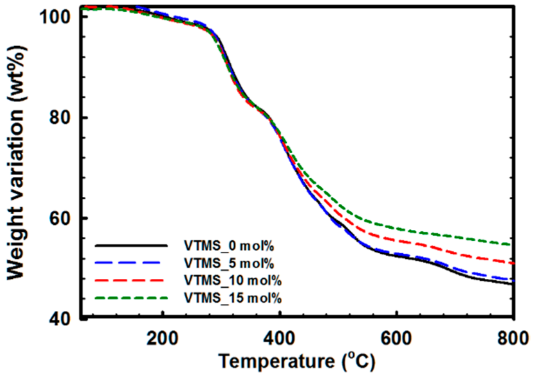

Figure S2. Thermal stability of P(SS-*co*-VTMS) with thermal annealing.

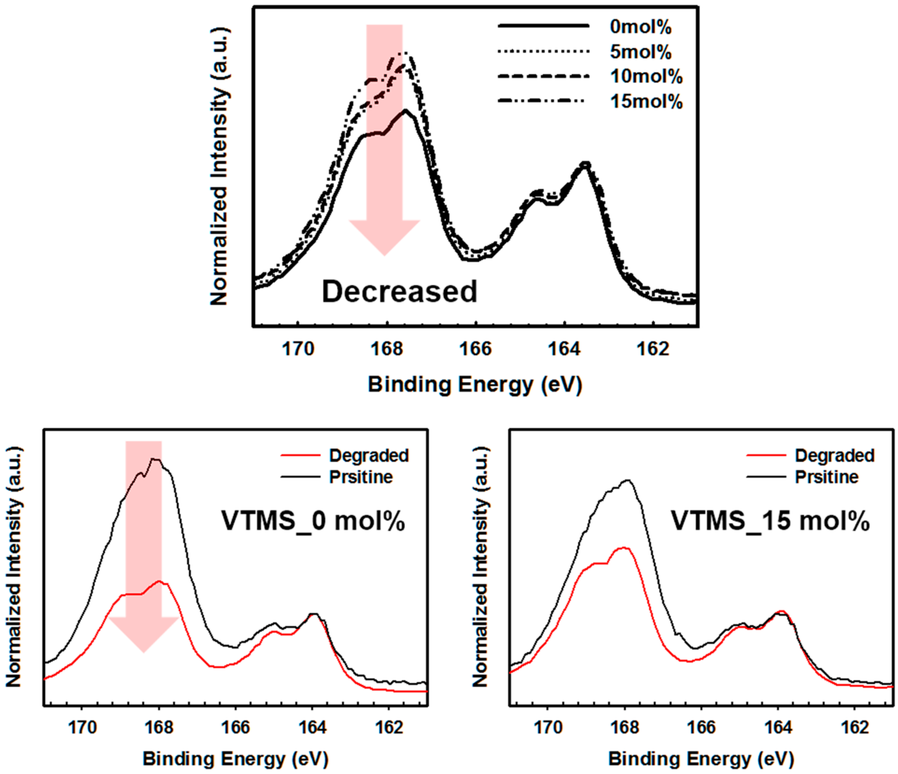

Figure S3. XPS spectra of PEDOT:P(SS-*co*-VTMS) films after thermal aging. The intensity of S 2p peaks at PSS, normalized with the S 2p peaks of PEDOT, was dramatically decreased as the VTMS_0 mol % conditions, however the intensity of S 2p peak at P(SS-*co*-VTMS) were relatively remained. The stability of conducting film dependent on the remain of S 2p peak at PSS.

© 2016 by the authors; licensee MDPI, Basel, Switzerland. This article is an open access article distributed under the terms and conditions of the Creative Commons Attribution (CC-BY) license (http://creativecommons.org/licenses/by/4.0/).
